# Model of Fear of Movement/(Re)injury Runs Clockwise From Catastrophizing: Evidence From a Sample of Outpatients With Chronic Nonspecific Low Back Pain

**DOI:** 10.1097/PHM.0000000000002659

**Published:** 2025-01-13

**Authors:** Marco Monticone, Federico Arippa, Luca Frigau, Calogero Foti, Silvano Ferrari, Marco Guicciardi, Barbara Rocca

**Affiliations:** From the Department of Surgical Sciences, University of Cagliari, Cagliari, Italy (MM); Department of Medical Sciences and Public Health and Department of Mechanical, Chemical and Materials Engineering, University of Cagliari, Cagliari, Italy (FA); Department of Economics and Business Sciences, University of Cagliari, Cagliari, Italy (LF); Department of Clinical Sciences and Translational Medicine, Tor Vergata University, Rome, Italy (CF); Department of Biomedical Sciences, Humanitas University, Pieve Emanuele, Milan, Italy (SF); Department of Education, Psychology and Philosophy, Faculty of the Humanities, University of Cagliari, Cagliari, Italy (MG); and Department of Clinical Psychology, International Institute of Behavioural Medicine, Seville, Spain (BR).

**Keywords:** Rehabilitation, Chronic Pain, Low Back Pain, Kinesiophobia, Catastrophization

## Abstract

**Objective:**

The aim of the study is to provide evidence that catastrophizing is the primer of the cognitive-behavioral model of fear of movement/(re)injury.

**Design:**

A cross-sectional analysis of 180 outpatients with chronic nonspecific low back pain who completed the Pain Catastrophizing Scale, the Tampa Scale of Kinesiophobia, the Roland-Morris Disability Questionnaire, the Hospital Anxiety and Depression Scale–Depression, and a pain intensity numerical rating scale. The intercorrelations of the outcome measures were estimated using Pearson’s correlation coefficient (r), and regression analyses were used to examine their predictive values by following the left side of the fear of movement/(re)injury clockwise from the Pain Catastrophizing Scale (*P* = 0.05). A postdictive analysis dichotomizing the sample into high- and low-level catastrophizers evaluated the effects of addressing catastrophizing on disability and pain.

**Results:**

The intercorrelations of the Pain Catastrophizing Scale with the Tampa Scale of Kinesiophobia, Roland-Morris Disability Questionnaire, Hospital Anxiety and Depression Scale–Depression and numerical rating scale were respectively r = 0.59, r = 0.54, r = 0.18, and r = 0.44. Pain Catastrophizing Scale was a significant predictor of Tampa Scale of Kinesiophobia; Pain Catastrophizing Scale and Tampa Scale of Kinesiophobia significantly predicted Roland-Morris Disability Questionnaire and Hospital Anxiety and Depression Scale–Depression; and Pain Catastrophizing Scale, Tampa Scale of Kinesiophobia, Roland-Morris Disability Questionnaire and Hospital Anxiety and Depression Scale–Depression significantly predicted numerical rating scale. The postdictive analysis showed that addressing catastrophizing reduces disability and pain experience by 14% in high-level catastrophizers and 86% in low-level catastrophizers.

**Conclusions:**

Our findings provide evidence that catastrophizing is the primer of the fear of movement/(re)injury.

**What Is Known**

- Catastrophizing principally traps people with chronic nonspecific low back pain in a vicious circle of mental and physical difficulties that includes fear avoidance, disability, depression, and pain experiences.

**What Is New**

- This study of outpatients with nonspecific low back pain verified that catastrophizing is the priming factor in the model of fear of movement/(re)injury and revealed the clockwise direction of the cycle of events after a back injury from catastrophizing to fear avoidance, disability, depression, and pain experience. It was also found that the impact of catastrophizing on disability and pain was different in the patients with high and low Pain Catastrophizing Scale scores.

Chronic low back pain (LBP) is a major disability,^1^ a serious, depressing, and undermining condition that has a painful impact on most of the subjects affected by it.^2^ The World Health Organization considers LBP to be chronic when it lasts for 12 wks or more^3^ but, although it is widespread, people find it surprisingly difficult to talk about.^4^

Catastrophizing has been defined as *an exaggerated negative orientation toward actual or anticipated pain experiences.*^5^ It reflects a predisposition to misinterpret or exaggerate outwardly threatening circumstances that may lead to increased sensitivity and enmesh sufferers in a vicious circle of mental and physical difficulties.^6^ Catastrophizing is also a marker of the greater use of rumination and blame attribution, and the less frequent use of cognitive reappraisal strategies. Its behavioral manifestations are associated with greater expressive suppression and withdrawal from or rejection of others,^5^ and it both chronicles and contributes to the persistence of LBP.^6^

As indicated in the model of fear of movement/(re)injury (FAM) published by Vlaeyen et al. in 1995,^7^ postinjury catastrophizing is followed by psychophysical reactions such as a fear of pain/movement, disability, depression, and pain experiences. More specifically, people with LBP go circularly from catastrophizing (the primer of maladaptive thoughts due to negative affectivity and erroneous information about their condition) to fear avoidance and disability and depression (at the same level), all of which contribute to the persistence of the pain.^7^

Chronic LBP requires adequate medical care,^8^ whereas catastrophizing requires psychological therapy.^9^ Both conditions worsened during the COVID-19 pandemic.^10,11^

The aim of this study was to provide further evidence of the role of catastrophizing in priming the fear avoidance, disability, depression, and pain experiences included in the FAM. Our working hypotheses were: i) catastrophizing correlates with fear avoidance, disability, depression, and pain experience in that order; ii) catastrophizing predicts fear avoidance, depression, disability, and pain experience; and iii) high- and low-level catastrophizing affect disability and pain differently.

## METHODS

### Participants

This is a secondary analysis of a previous single-group, cross-sectional investigation aimed at evaluating the Pain Catastrophizing Scale (PCS) in outpatients with chronic nonspecific LBP who were referred to a rehabilitation hospital and three affiliated centers by their general practitioners between January and June 2010.^12^ This study was approved by the institutional review board of Salvatore Maugeri Foundation’s Scientific Institute of Lissone, Italy (IRB approval number 08/09). Patients gave their written consent to take part in the study.

The enrolled patients had to be adults with chronic nonspecific LBP^3^ who were capable of reading and speaking Italian fluently. The inclusion criteria were a diagnosis of chronic nonspecific LBP (i.e., documented history of pain lasting >3 mos in the absence of any diagnosable cause), a good understanding of Italian, and being adult. The exclusion criteria were acute and subacute nonspecific LBP; definitely known causes of specific LBP; central or peripheral neurological signs; systemic illnesses or psychiatric deficits; a recent myocardial infarction or cerebrovascular event; and chronic lung or renal disease. People who underwent spinal surgery were excluded. The eligible patients were told of the purposes and methods of the study and required to give their informed consent; none of them refused to participate. Two hundred-eight people were screened, of whom 28 were excluded because of specific causes of nonspecific LBP (*n* = 11), cognitive impairment (*n* = 3), recent myocardial infarction (*n* = 1), systemic illness (*n* = 2), recent cerebrovascular event (*n* = 1), while 10 had personal problems. The study involved 180 patients with chronic nonspecific LBP: 77 women (43%) and 103 men (57%) whose mean age was 44.1 ± 11.3 years (range 18–73). The median duration of nonspecific LBP was 12 mos (range 3–48). The clinical characteristics were ruled out by case history and lumbar radiographs; in doubtful cases, we adopted computed tomography or magnetic resonance imaging.^12^ See Table 1.

**TABLE 1:** Clinical and Sociodemographic characteristics of the sample (*N* = 180)

Age (Years), Mean ± SD		44.1 ± 11.3
Gender (*n*)	Male	103 (57%)
	Female	77 (53%)
Marital status (*n*)	Married	106 (58.9%)
	Unmarried	74 (41.1%)
Education level (*n*)	Primary school	10 (5.6%)
	Middle school	20 (11.1%)
	High school	77 (42.7)
	University	73 (40.6%)
Employment	Dependent employee	115 (63.9%)
	Self-employed	65 (36.1%)
Smokers (*n*)	Yes	43 (23.9%)
	No	137 (76.1%)
Medications	Antidepressants	16 (8.9%)
	Analgesics	74 (41.2%)
	Muscle relaxants	20 (11.1%)
	NSAIDs	70 (38.8%)
Comorbidities (*n*)	Hypertension	57 (31.6%)
	Noninsulin-dependent diabetes mellitus	23 (12.7%)
	Heart disease (not acute)	10 (5.6%)
	Enteric disease (not acute)	11 (6.1%)
	Liver disease (not acute)	5 (2.8%)
	None	74 (41.2%)
Body mass index (kg/m^2^), mean ± SD		24.2 ± 3.7

NSAIDs, non-steroidal anti-inflammatory drugs.

The study complied with all of the national regulations and institutional policies concerning research involving human subjects, as well as the tenets of the 2013 amendment of the Helsinki Declaration.

### Outcome Measures

The Italian version of the PCS, a 13-item self-administered questionnaire that asks respondents to indicate the degree to which they have had each of 13 thoughts or feelings during previous experiences of pain using 5-point Likert scales ranging from 0 (not at all) to 4 (all the time). The total score is the sum of the scores of the individual items (range 0–52), with higher scores indicating greater catastrophizing.^6^ It has been shown that the Italian version is satisfactorily reliable (internal consistency: 0.77; test-retest reliability: 0.95).^12^

The Italian version of the Tampa Scale of Kinesiophobia (TSK), a 13-item self-administered questionnaire that asks respondents to indicate the extent to which they agree or disagree with each statement using 4-point Likert scales ranging from 1 (strongly disagree) to 4 (strongly agree). The total score is calculated by adding the scores of the individual items (range 13–52, with 52 indicating the worst catastrophizing). It has been shown that the Italian version is satisfactorily reliable (internal consistency: 0.92; test-retest reliability: 0.84).^13^

The Italian version of the Roland-Morris Disability Questionnaire (RMDQ), a 24-item self-administered questionnaire that asks respondents to indicate the items applicable to them on that day. The items are scored 1 if considered applicable and 0 if not, and so the total score may range from 0 (no disability) to 24 (severe disability). It has been shown that the Italian version is satisfactorily reliable (internal consistency: 0.82; test-retest reliability: 0.92).^14^

The Italian version of the Hospital Anxiety and Depression Scale (HADS), a 14-item self-administered instrument that asks respondents to rate the degree to which each item describes how they have been feeling over the previous week using a 4-point Likert scale ranging from 0 to 3. For the purposes of this study, only the depression subscale (HADS-D) was used, and so the total score (the sum of the scores of the seven individual items) ranged from 0 to 21, with higher scores indicating greater depression.^15^ It has been shown that the Italian version is satisfactorily reliable (internal consistency: 0.85).^16^

A self-administered numerical rating scale (NRS) consisting of a horizontal line divided into 11 segments numbered 0 (no pain) to 10 (the worst possible pain).^17^

### Statistical Analysis

The intercorrelations between outcome measures estimated by means of Pearson’s correlation coefficient (r) can provide insights into the strength and direction of the relationships between variables. In the context of the fear avoidance model,^7^ the expected intercorrelations between the variables can shed light on the potential associations predicted by the theoretical framework,^7^ with the PCS correlating more closely in magnitude and direction to the TSK than the RMDQ than the HADS-D than the NRS. Correlations were considered low if r was <0.3, moderate if 0.3 < r < 0.5, and high if r was >0.5.^18^

Multivariate hierarchical regression analysis was used to confirm the circular events described by the FAM^7^ by investigating the predictive role of catastrophizing on fear avoidance, disability, depression, and pain experience. A distinctive feature of this study was that the variables used to assess catastrophizing, depression, and pain were different from those used by the authors of the original study (i.e., the Pain Cognition List, the Beck Depression Inventory, and a visual analog scale); furthermore, we used the RMDQ to assess disability, whereas Vlaeyen et al. did not use any measure to evaluate this construct.^7^ The predicted factors were iteratively included in the successive steps of the model: for example, the PCS was first introduced as a predictor of TSK, then the PCS and the TSK were used as predictors of the RMDQ and the HADS-D (which were introduced at the same level), and so on. Pain duration and gender were entered in the model as confounding factors. The set level of significance was *P* = 0.05.

A postdictive analysis^19^ was made by dichotomizing the sample into high- and low-level catastrophizers^20^ and cross-referencing the RMDQ and NRS values divided into quartiles.

All of the analyses were made using SPSS v.29.

## RESULTS

The intercorrelations of the PCS with the TSK, the RMDQ, the HADS-D and the NRS were respectively r = 0.59 (*P* < 0.001), r = 0.54 (*P* < 0.001), r = 0.18 (*P* = 0.017), and r = 0.44 (*P* < 0.001).

The results of the hierarchical regression analysis showed that the PCS was a significant predictor of the TSK and explained 35% of the variance (*P* < 0.001, R^2^ = 0.35). The PCS and the TSK significantly predicted the RMDQ and the HADS-D and respectively explained 42% and 6% of the variance (*P* < 0.001, R^2^ = 0.42; *P* = 0.026, R^2^ = 0.06). The PCS, TSK, RMDQ, and HADS-D significantly predicted the NRS and explained 54% of the variance (*P* < 0.001, R^2^ = 0.54).

The descriptive postdictive findings of the cross-referenced disability and pain quartiles showed that most of the cases fell into the category of low-level catastrophizers (i.e., PCS <30). See Tables 2 and 3 for the full estimates.

**TABLE 2:** Postdictive analysis of PCS scores (as dichotomized according to Wertli et al.^21^) cross-referenced against the quartiles of the RMDQ scores

RMDQ				
	Q1 = (0–3)	Q2 = (4–6)	Q3 = (7–11)	Q4 = (12–23)
PCS < 30	43 (24%)	45 (25%)	42 (23%)	25 (14%)
PCS ≥ 30	3 (2%)	2 (1%)	6 (3%)	14 (8%)

Q1, 25% quartile; Q2, 50% quartile; Q3, 75% quartile; Q4, 100% quartile.

**TABLE 3:** Postdictive analysis of PCS scores (as dichotomized according to Wertli et al.^21^) cross-referenced against the quartiles of the NRS

NRS				
	Q1 = (0–3)	Q2 = (4)	Q3 = (5–6)	Q4 = (7–10)
PCS < 30	63 (35%)	34 (19%)	42 (23%)	16 (9%)
PCS ≥ 30	5 (3%)	2 (1%)	4 (2%)	14 (8%)

Q1, 25% quartile; Q2, 50% quartile; Q3, 75% quartile; Q4, 100% quartile.

## DISCUSSION

The first of our working hypotheses was partially confirmed, and the other two were confirmed: catastrophizing correlated with the other measures but in a different order from that expected (fear avoidance, disability, pain experience, and depression); catastrophizing predicted fear avoidance, depression, disability, and pain experience; and there were differences in the impact of catastrophizing on disability and pain between high- and low-level catastrophizers.

The intercorrelations between catastrophizing and the other measures were moderate to low. As expected, the highest correlation was with fear avoidance: this was no surprise as catastrophizing is the primer of the maladaptive thoughts that elicit the model of fear of movement/(re)injury in people with backache.^21^ Catastrophizing induces fear avoidance, and people with chronic nonspecific LBP need multidisciplinary treatments that target these maladaptive thoughts in order to have a chance of achieving enduring results.^22,23^ Catastrophizing also correlated with disability, but to a slightly lesser extent. This too was not surprising because most catastrophizers due to chronic nonspecific LBP are also disabled.^2^ People with chronic nonspecific LBP therefore need multidisciplinary treatments that incorporate the management of physical impairment.^22^ The correlation between catastrophizing and pain experience was lower probably because the consolidation of common chronic complaints means that patients perceive pain as less unpleasant than their fears of spinal movement or impairment during everyday activities.^24^ On the other hand, we were surprised to find that the lowest correlation was between catastrophizing and depression because others have generally found a moderate correlation.^24^ However, it is known that catastrophizing is favored by anxiety, and this may explain the low correlation with depression.^25^

The hierarchical regression analysis revealed that catastrophizing predicts fear avoidance, which, in its turn, predicts disability and depression, and all together predict pain.^7^ Our findings support the hypothesis that the molehill that people with chronic pain perceive as a mountain (i.e., catastrophizing) is actually the priming emotion.^26^ Catastrophizers have a restricted view of their problem due to the fusion of their thoughts with the presence of illness behaviors (e.g., the avoidance of everyday activities avoidance, maladaptive comparisons with others, and poor personal care), and these factors undermine improvements in mood disorders and the management of sorrow.^27^ Interestingly, the use of some different variables from those used by Vlaeyen et al.^7^ and the introduction of a measure of disability did not interfere with the circularity of the model of fear of movement/(re)injury, but may even extend its applicability to contexts of motor rehabilitation. Given its components of helplessness, rumination, and magnification, catastrophizing assumes the characteristic of a maladaptive coping strategy. As such, it must be identified promptly as part of a comprehensive assessment to break the vicious cycle into which persons with chronic LBP may fall down, including the relationships with fear avoidance behaviors.^7^ Furthermore, exhibiting these psychological features, catastrophizing can only be treated by a specialist in the field using techniques that pursue SMART (i.e., specific, measurable, achievable, relevant, and time-based) objectives, graded exposure, pacing, cognitive restructuring, systematic desensibilization, etc.

Our postdictive analysis could be made because it was possible to dichotomize our sample into high- and low-level catastrophizers. It is crucial to be able to distinguish high-level catastrophizers (those with a PCS score of ≥30) as they have a greater need for psychological care,^21^ and our analysis showed that addressing this need could lead to an approximately 14% in the number of people with chronic nonspecific LBP experiencing disability and pain. However, it was particularly interesting to find that targeting low-level catastrophizers could lead to an 86% reduction, which is epidemiologically and clinically even more important and should never be neglected by healthcare personnel. It goes without saying that, regardless of their level of catastrophizing, all catastrophizers should be treated in order to reduce disability and pain in them all. The relatively low cost of multidisciplinary treatments that include psychological and exercise-based interventions (€ 230 per patient according to a previous study carried out in Italy^28^) in comparison with other treatments^29^ means that they may be the best approach for people with chronic nonspecific LBP as they also save the number of days lost from everyday living activities and contribute to avoiding noneffective treatments.

This study has a number of limitations. It did not investigate the effect of catastrophizing on or its relationships with physical tests. The sample of outpatients with chronic nonspecific LBP came from rehabilitation facilities, and the findings of the study cannot be generalized to other contexts or conditions; furthermore, the patients were the same as those used to validate the Italian version of the PCS whereas two different samples may have been preferrable. Moreover, catastrophizing is probably very culturally dependent and, as the study was performed in Italian persons, our findings may not be generalizable to other populations. The study was cross-sectional and, although significant, the correlations and regression weights should not be confounded with causal associations; this also prevented us from adopting a structural equations model that would have allowed us to provide firm evidence concerning the causal relationships among the variables. Data on specific home exercise programs were not reported and this prevented us from sharing this information. Finally, postdictive analyses should be regarded with caution.

In conclusion, this study provides further evidence that catastrophizing primes the model of fear of movement/(re)injury. Catastrophizing intercorrelated with fear avoidance, disability, pain experience, and depression and predicted them all. The impact of catastrophizing on disability and pain experience was different between high- and low-level catastrophizers.

### Clinical and Rehabilitative Implications of the Findings

Pain management should not be regarded as the primary target when addressing common chronic low back complaints. When subjects become catastrophizers and fear avoiders, and really experience what feeling endless pain is, it is important to rely on back-care specialists (spinal physicians, physiotherapists, occupational therapists, and psychologists).^30^

Multidisciplinary rehabilitation treatments including effective physical and psychological interventions should be considered first-line treatment options for people with chronic nonspecific LBP^1^ and can save costs over time.

In more details, people who have suffered from nonspecific LBP for a long time need to reduce maladaptive thoughts and irrational beliefs on pain, acquire new notions about backache, learn novel management strategies during daily tasks, and implement gradual recovery of activities. In this regard, cognitive-behavioral therapy represents a valid therapeutic option.

### Research Agenda

- Provide further evidence that people with chronic nonspecific LBP adopt withdrawal behaviors.
- Examine the value of considering catastrophizing as the emotional primer when planning multidisciplinary treatments for people with chronic nonspecific LBP that include exercise and psychological interventions.
- Compare the costs of multidisciplinary rehabilitative treatments of chronic nonspecific LBP with those of other approaches.
- Verify the possibility of further enhancing the clinical impact of the fear avoidance model by adding anticlockwise reverse tracking starting with pain, which is the hub of the model.
- Perform studies involving brain functional magnetic resonance imaging to compare changes in the different individuals’ catastrophizing/kinesiophobia categories.
